# Effects of Wood Flour (WF) Pretreatment and the Addition of a Toughening Agent on the Properties of FDM 3D-Printed WF/Poly(lactic acid) Biocomposites

**DOI:** 10.3390/molecules27092985

**Published:** 2022-05-06

**Authors:** Wangwang Yu, Mengqian Li, Wen Lei, Yongzhe Pu, Kangjun Sun, Yilong Ma

**Affiliations:** 1 College of Science, Nanjing Forestry University, Nanjing 210037, China; yuww@niit.edu.cn (W.Y.); tougao9a318@163.com (M.L.); puyongzhell@163.com (Y.P.); www.qq2414443695@163.com (K.S.); www.qq1104459062@163.com (Y.M.); 2 School of Mechanical Engineering, Nanjing Vocational University of Industry Technology, Nanjing 210023, China

**Keywords:** poly(lactic acid) (PLA), wood flour (WF), composite, silane coupling agent, acetic anhydride, acrylicester resin, fused deposition modeling (FDM)

## Abstract

In order to improve the properties of wood flour (WF)/poly(lactic acid) (PLA) 3D-printed composites, WF was treated with a silane coupling agent (KH550) and acetic anhydride (Ac_2_O), respectively. The effects of WF modification and the addition of acrylicester resin (ACR) as a toughening agent on the flowability of WF/PLA composite filament and the mechanical, thermal, dynamic mechanical thermal and water absorption properties of fused deposition modeling (FDM) 3D-printed WF/PLA specimens were investigated. The results indicated that the melt index (MI) of the specimens decreased after WF pretreatment or the addition of ACR, while the die swell ratio increased; KH550-modified WF/PLA had greater tensile strength, tensile modulus and impact strength, while Ac_2_O-modified WF/PLA had greater tensile modulus, flexural strength, flexural modulus and impact strength than unmodified WF/PLA; after the addition of ACR, all the strengths and moduli of WF/PLA could be improved; after WF pretreatment or the addition of ACR, the thermal decomposition temperature, storage modulus and glass transition temperature of WF/PLA were all increased, and water absorption was reduced.

## 1. Introduction

Three-dimensional printing, a subset of additive manufacturing, has experienced rapid development in recent years and attracted various types of industries throughout the world [1,2], it can be used to fabricate near-net-shaped complex 3D parts without expensive molds or tools in short periods of time, and it enables designers to build real objects accurately by computer-aided design [3,4,5]. Several 3D printing techniques have been developed for fabricating thermoplastic polymers and composites; printing based on fused-deposition modeling (FDM) is particularly widespread because of its low-cost equipment, variability in material selection and easy operation [5,6,7].

At present, the usage of a suitable material is still one of the key aspects affecting the development of 3D printing technology, many efforts have been made to develop new materials for this technology [8] and the polymers, such as polylactic acid (PLA), acrylonitrile-butadiene-styrene copolymer (ABS) [9], polyamide(PA) [10], polybutyrate-adipate-terephthalate (PBAT) [11], polycaprolactone (PCL) [12], poly(butylene succinate) (PBS) [13], poly(hydroxyalkanoate) (PHA) [14] and thermoplastic polyurethane (TPU) [4], have been reported for FDM 3D printing. PLA, a biodegradable polymer that comes from renewable resources with favorable mechanical properties, low elongation at break, low thermal expansion coefficient, good melt processability and reproducibility [3,7,15], is the most popular material. It can be simply hydrolyzed without additional enzymes and has no health risk to humans as compared to the ABS when used in nonventilated areas. Even so, its high price limits the wider application of PLA’s FDM technology.

Wood flour (WF), a by-product of the wood processing industry, has the advantages of low density, high modulus, biodegradability, low abrasion, as well as low cost. Adding wood flour to the PLA may reduce the production cost of PLA (the unit price of WF is only about 2% of PLA in China, and the production cost can be saved by approximately 1% when 1wt.% WF is used in PLA), endow the printed items with woodiness and decrease weight loss. The (FDM) 3D-printed WF/PLA can be manufactured into a variety of products, such as works of art, teaching models, lamp frames and pen containers. Thus, the use of WF/PLA composites as the filament for 3D printing has grown in recent years [3]. However, the interfacial compatibility between wood flour and PLA is poor due to their polarity differences. In order to obtain high-performance WF/PLA composites, the interface between WF and PLA is critical. Just like that in other natural fiber-reinforced composites, the modification should be carried out to make sure reinforcements have good interfacial interaction with the polymer matrix in terms of physical absorption, chemical absorption, electrostatic interaction and mechanical connection. The ways to improve interfacial adhesion include fiber pretreatment and additive addition [16]. Rui Guo et al. [17] studied the effect of toughening agents (TPU, PCL and polyolefin elastomer (POE)) on the properties of the FDM 3D WF-reinforced PLA composite. It was found that the impact strength of composites was significantly increased with the addition of TPU but decreased with the addition of PCL or POE. Guoqiang Xie et al. [18] prepared WF/PLA filaments via a melt extrusion method; three kinds of plasticizing agents, i.e., 4% glycerol, 2%glycerol + 2%tributyl citrate (TBC) and 4%TBC were used, and the properties of 3D-printed specimens were comparatively investigated. The results showed that the 3D-printed sample with 4% glycerol had the highest MI, which indicated that glycerol was more favorable for the extrusion processing of composite filaments, while the sample with 4%TBC had the greatest tensile strength and the least water uptake. Additionally, it was the most thermally stable. Nasır Narlıoğlu [19] reported the modification of wood flour with butyric anhydride and its effect on the properties of 3D-printed WF/PLA composites. It was found that the interface bonding between WF and PLA was improved, and the tensile strength was increased. Qingfa Zhang et al. [20] utilized a lubricant (TPW604) and a toughening agent (POE) to improve the fluidity and toughness of WF/PLA composites, and they found that both TPW604 and POE could improve the fluidity of 3D printing materials, and POE could also effectively improve the impact strength of 3D printing materials.

In this work, two different chemical modifications (silane coupling agent treatment and acetic anhydride treatment) for WF and one toughening agent (acrylicester resin (ACR)) were applied to improve the compatibility between fiber and polymer. The effects of the chemical modifications on the chemical structures of WF were studied by infrared spectroscopy (IR), and the effects of WF modifications and application of the toughening agent on melt flowability and dimensional swell of the composite filaments were first discussed, then on mechanical, dynamic mechanical and thermal properties of the printed composites were studied. Additional emphasis was placed on the water absorption of the composites.

## 2. Results and Discussion

### 2.1. Fourier Transform Infrared Spectroscopy

**Figure 1:**
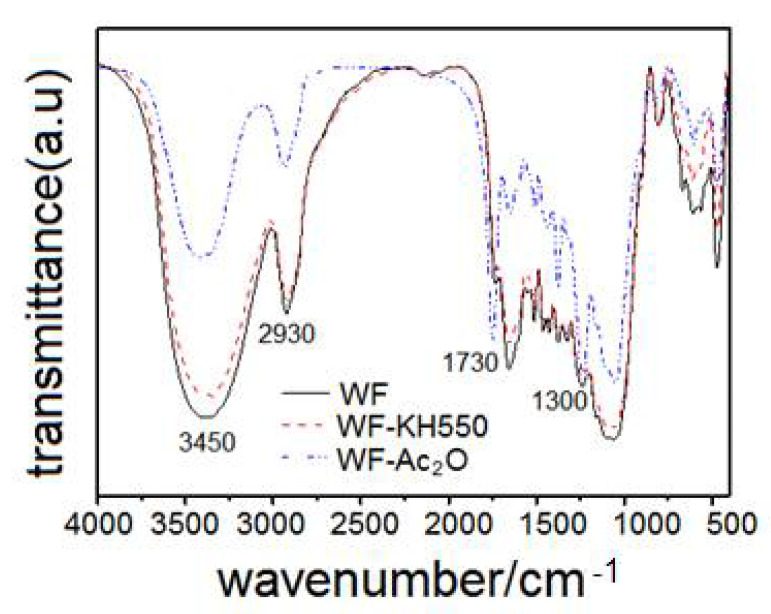
FTIR spectra of WF before and after pretreatment.

The FTIR spectra of virgin WF, WF-KH550 and WF-Ac_2_O are shown in Figure 1. In the untreated WF spectrum, the broad peaks at 3450 cm^−1^ and 2930 cm^−1^ are attributed to the O-H stretching vibration of hydrogen-bound hydroxyl groups of cellulose and hemicellulose [21] and the C-H stretching vibration of alkyl groups [15], and the distinctive symmetric peaks at 1730 cm^−1^ and around 1300 cm^−1^ correspond to the C=O carbonyl stretching [22] and the C-O stretching vibration (nonconjugated), respectively [23]. Spectra for WF-KH550 and WF-Ac_2_O were similar to the neat WF, but for WF-Ac_2_O, the relative intensity of these features at 1730 cm^−1^ and around 1300 cm^−1^ increased, which was attributed to the esterification of Ac_2_O and the –OH of WF. For WF-KH550, the position of C=O carbonyl stretching and C–O stretching vibration peaks had shifted because of the hydrogen bond interaction between the –OH of KH550 and the –OH of WF. The esterification and the hydrogen bond interaction could both improve the bonding between WF and PLA in the 3D printing materials.

### 2.2. Melt Index and Relative Die Swell Ratio

The composite filaments were prepared via a melt extrusion method under the condition of melt flow. The processed material should have proper flowability to ensure the production runs smoothly. A too-small flowability of the melt will make it difficult to fill the mold and hard to be extruded. On the contrary, a too-large flowability will make it difficult to form enough extrusion pressure, and as a result, the shaping and strength of the printed samples are negatively affected [18]. Therefore, it is necessary to assess the melt flowability of the composite filaments when WF is modified or ACR is used.

**Figure 2:**
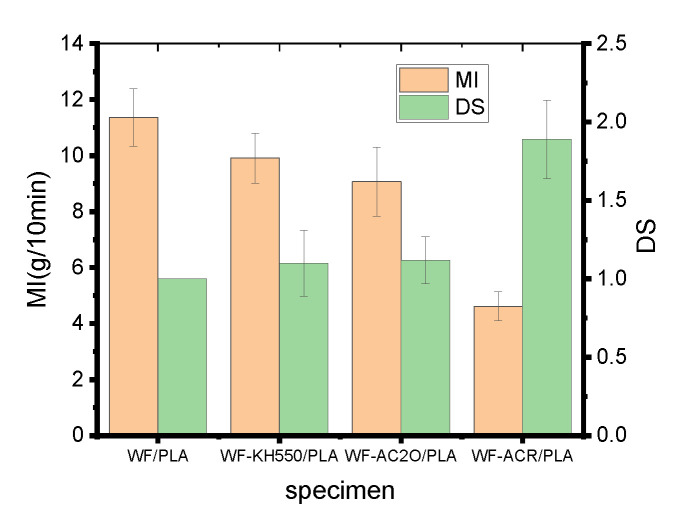
Melt index (MI) and relative melt die swell ratio of composite filaments.

Figure 2 indicates the effects of WF pretreatments and the addition of a toughener on the MI of 3D-printed materials. As shown in Figure 2, WF pretreatments and the addition of ACR all reduced the MI of the composites. The MI reduced from 11.36 g/10 min for virgin WF/PLA to 9.91 g/10 min for WF-KH550/PLA and 9.07 g/10 min for WF-Ac_2_O/PLA. In particular, for WF/PLA-ACR, the MI decreased dramatically to 4.62 g/10min, which is because ACR has a great effect on the flexibility of WF/PLA, so when it is used, the melt elasticity becomes greater during the extrusion process, which leads to the poor fluidity of composites. Even so, the MI of the specimens can all meet the requirements for processing.

The effect of the WF pretreatment and the addition of ACR on the flowability of the composite can also be proved by the results of the relative die swell ratio as also shown in Figure 2. All the modified composite filament had greater die swell ratios than unmodified WF/PLA composite filament. This means that the flowability of the composite became poorer after the modification, which was consistent with the results of the MI testing. After the modification, the interfacial bonding between WF and PLA becomes stronger; it becomes more difficult for the melts to flow; the residence time of the polymer melting in the die during extrusion flow increases; and the elastic recovery of the shear deformation and elongation deformation generated during polymer melt extrusion flow will be increased after the melt leaves the die [24], leading to an increase in the melt die swell ratio.

### 2.3. Mechanical Property

**Figure 3:**
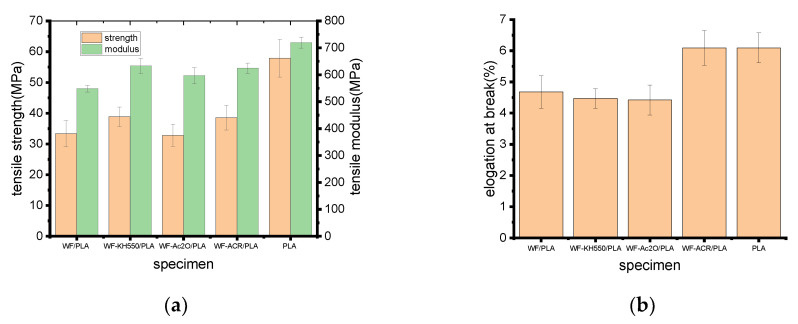

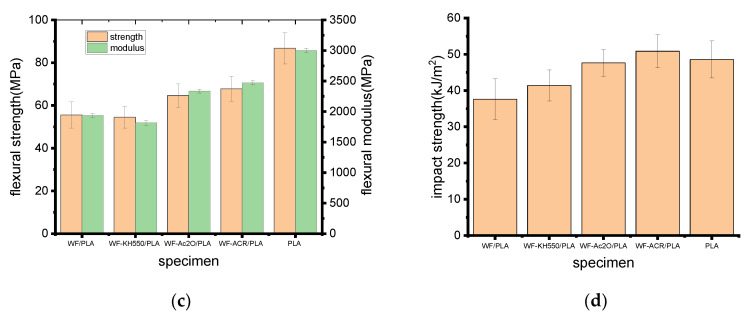
Mechanical properties of the printed materials: (**a**) tensile strength and modulus; (**b**) elongation at break; (**c**) flexural strength and modulus; (**d**) impact strength.

Tensile properties of various printed samples are shown in Figure 3a,b. It could be seen that adding WF to PLA generally decreased the tensile strength and modulus of the resin, and the reduced tensile strength may be due to the weak interface bonding between wood flour/plant fiber and PLA [19,25]. As a result of the comparison of the tensile strengths and moduli of the composites, it was seen that the tensile strength was higher for WF-KH550/PLA or WF-ACR/PLA but a tiny lower for WF-Ac_2_O/PLA compared to WF/PLA. The tensile modulus of WF-KH550/PLA, WF-Ac_2_O/PLA and WF-ACR/PLA was increased by 15.35%, 8.75% and 13.83%, respectively, indicating that after WF pretreatment or the addition of ACR, good interaction occurred between WF and the PLA matrix, resulting in good dispersion of fiber materials across the matrix and led to an enhancement in the material stiffness [21]. The elongation at the break of the composites decreased by around 5% after WF pretreatment. This may be due to the restriction of molecular mobility with pretreated WF in the matrix. However, 30.13% of elongation at the break was increased for WF-ACR/PLA as compared to WF/PLA. In this situation, the elongation at the break of the composite was quite near that of neat PLA. This indicated that ACR made the composites toughened.

Flexural properties of the printed biocomposites of WF/PLA, WF-KH550/PLA, WF-Ac_2_O/PLA and WF-ACR/PLA are shown in Figure 3c. Meanwhile, those of neat PLA are illustrated as a reference. Just like that for the tensile properties, the addition of WF also reduced the flexural strength and modulus of PLA, which is consistent with the results of some reported literature [19,26]. When the flexural properties of the printed composites were considered, it can be seen that WF-ACR/PLA samples exhibited the highest flexural strength of 67.83 MPa and flexural modulus of 2470.20 MPa, which were 22.11% and 27.56% higher than those of virgin WF/PLA composites, respectively. The WF-Ac_2_O/PLA composite also had much greater flexural strength and modulus than WF/PLA, while the flexural properties of the WF-KH550/PLA were slightly poorer than those of WF/PLA, indicating that the effects of WF pretreatments on the flexural properties of the composites are not exactly the same as those on the tensile properties. This is because the flexural samples fail in combination of compression, shear and tension mode [27].

Figure 3d shows the impact strengths of all the studied specimens. Overall, the results show that all the modified WF/PLA gave higher impact strengths than the original WF/PLA, among which the addition of ACR improved the impact strength of the composites, most obviously by 35.17%, which was even greater than that of neat PLA by 4.72%. Concerning WF/PLA, the known chemical incompatibility between WF and PLA can explain the lower impact strength observed compared to those obtained with WF-KH550, WF-Ac_2_O or ACR. This chemical incompatibility induces poor interfacial bonding between WF and PLA.

### 2.4. Thermal Stability

**Figure 4:**
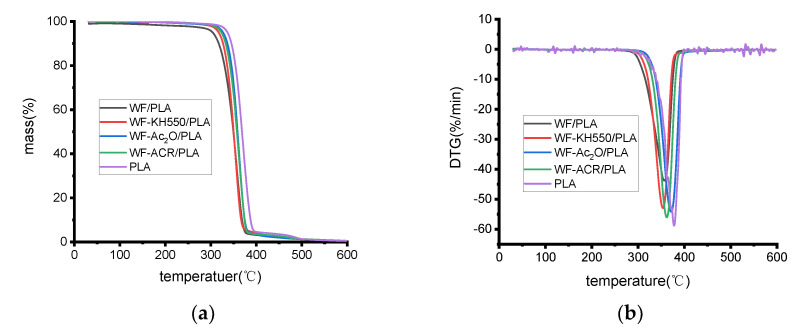
(**a**) TGA curves and (**b**) DTG curves for the samples indicated at 20 K/min in a nitrogen atmosphere.

Figure 4 shows the thermogravimetric (TGA) and first derivative thermogravimetric (DTG) curves of neat PLA and various composites. The thermal stabilities of the samples were characterized by the temperature at which the initial decomposition occurred, i.e., the onset temperature (T_i_), and the temperature at which the maximum rate of weight loss occurred (T_p_). The respective thermal properties for the main peaks are given in Table 1. As depicted in Figure 4 and Table 1, neat PLA indicated initial decomposition and the degradation peak at 334.5 °C and 375.8 °C, respectively, which are very close to the reported temperatures of 337.8 °C and 369.2 °C in the literature [1], while WF/PLA resulted in 307.8 °C and 356.1 °C for when initial degradation occurred and when the peak shifted to lower temperatures, and a similar trend has been detected for the coir/PLA composites [28] and kenaf/PLA [1]. This can be explained by the associative thermal stability with the hindered diffusion of the decomposition products [29]. When WF is incorporated with PLA, the decomposition products may become easier to diffuse because of the increased pathway of degradation, leading to a lowered onset temperature, and the interaction between molecules becomes weakened; consequently, less heat is required to break the interaction between WF and PLA, and the thermal stability of the printed specimens thus becomes poorer [1].

After WF pretreatment or the addition of ACR, the thermal stabilities of the composites could be improved, WF-KH550/PLA, WF-Ac_2_O/PLA and WF-ACR/PLA all became to decompose at much higher temperatures than WF/PLA and the char residue obtained with WF-KH550/PLA, WF-Ac_2_O or WF-ACR/PLA was all more than 20% higher char residue compared to WF/PLA. In this study, it is speculated that the degradation of modified WF/PLA at higher temperatures and with more residue can be attributed to the reduced free hydroxyl groups after chemical modification [19]. The increase in thermal stability means that more energy will be needed to damage the adhesion during the process of degradation [18]. This again confirms that the WF pretreatment and the addition of ACR improved the interfacial bonding between WF and PLA, which is also consistent with those from FTIR analysis and mechanical property testing.

### 2.5. Dynamic Mechanical Properties

**Figure 5:**
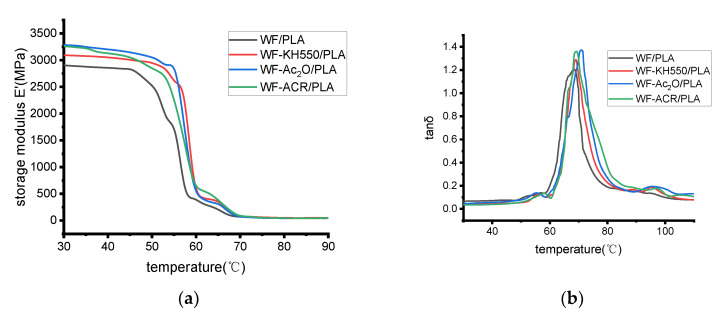
(**a**) Storage modulus and (**b**) loss angle tangent as a function of temperature for different printing samples.

Dynamic mechanical analysis was performed on WF/PLA, WF-KH550/PLA, WF-Ac_2_O/PLA and WF-ACR/PLA, respectively. The curves of storage modulus(E’) and tan delta (tan δ) vs. temperature are shown in Figure 5a,b, respectively. The glass transition temperature (T_g_) was obtained from the peak temperature of tan delta (tan δ) in the curves shown in Figure 5b and provided in Table 2.

At 30 °C, the WF/PLA specimen showed a storage modulus of 2906.30 MPa, and the storage modulus increased to 3095.17 MPa for WF-KH550/PLA, 3287.26 MPa for WF-Ac_2_O/PLA and 3262.32 MPa for WF-ACR/PLA by 6.50%, 13.11% and 12.25%, respectively. T_g_ values of WF-KH550/PLA, WF-Ac_2_O/PLA and WF-ACR/PLA were all higher than that of WF/PLA. The T_g_ of WF-Ac_2_O/PLA was the highest one. This may be because when WF is treated with acetic anhydride, the contact between the modifier and the WF is the most complete, and the reaction between PLA and WF-Ac_2_O is more intensive, thus enhancing the restriction on the mobility of molecular chains.

### 2.6. Water Absorption

**Figure 6:**
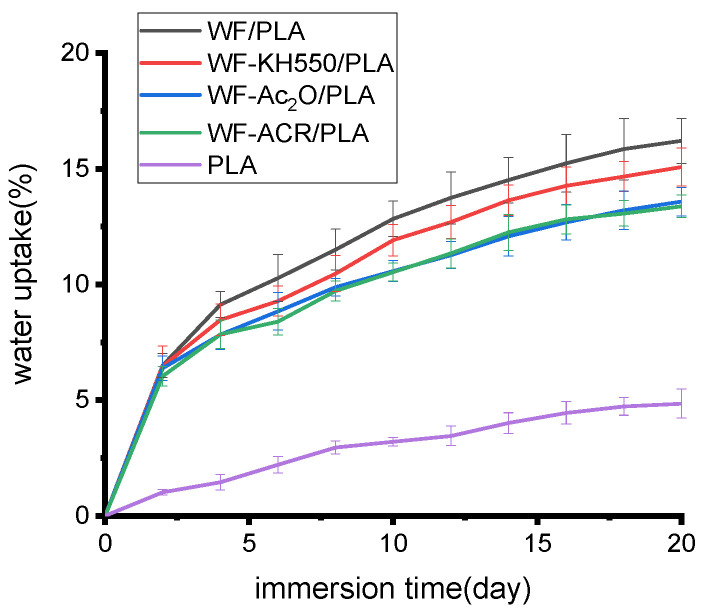
Effect of WF pretreatment and compatibilizer on water absorption of 3D-printed specimens with time.

Water absorption curves based on experimental results were developed for each of the specimen types of varying modifications and immersion times (see Figure 6). The water absorption analysis results of the composite specimens show that all the printed specimens with different pretreatments on WF or the addition of ACR differed from one another after being immersed in water, and they all always absorbed less water than the WF/PLA composite at the same immersion time. The obtained results for composites were similar to reported values obtained for some other natural fiber/polymer composites [30,31]. For WF/PLA, the fiber and matrix are mixed directly, the interfacial bonding is inferior and the specimen can absorb moisture better. When it is pretreated with KH550 or Ac_2_O, modified WF contained less exposed hydroxyl groups than original WF, and the interfacial bonding between WF and PLA is enhanced, resulting in a reduction in water absorption. When ACR is added, the compatibility between WF and PLA is improved, interfacial defects are reduced and, consequently, less moisture will be absorbed.

Compared with the neat PLA, all the composites absorbed more water at the same immersion duration. A high level of moisture absorption by natural fibers can reduce the mechanical properties and degradation temperature of the composites [32,33]. From the perspective of this study, adding WF to PLA made the water absorption of the printed specimen increase; however, WF treatment or the application of a toughening agent can be carried out to decrease the water absorption capacity of the composites. Therefore, it will help improve the mechanical and thermal properties of the 3D-printed specimen.

## 3. Experimental

### 3.1. Materials and Reagents

PLA(American Nature Works Co., 3052D, Minnetonka, MN, USA) was purchased from Shanghai Xingyun International Trade Co. Ltd., Shanghai, China; WF, 300 mesh, was obtained from Feixian Wood Fiber Factory, Linyi, China; 3-Aminopropyltriethoxysilane(KH550), CP, was obtained from Nanjing Shuguang Chemical Group, Nanjing, China; acetic anhydride(Ac_2_O), AR, was obtained from Linfeng Chemical Reagent Co. Ltd., Shanghai, China; acrylicester resin(ACR), apparent density between 0.4 g/cm^3^~0.55 g/cm^3^, 40 mesh, was obtained from Shandong Ruifeng Chemical Co. Ltd., Zibo, China.

### 3.2. Pretreatment of WF

Coupling agent pretreatment: The silane coupling agent KH550 was dissolved in 30wt% ethanol/water solution and kept hydrolyzed for 15 min to obtain the silane hydrolysate, and then wood flour was added to the hydrolysate, yielding a silane-to-WF ratio of 3:100. The mixed solution was further stirred for 2.5 h, filtered and washed several times with distilled water. The WF was then dried to obtain coupling-agent-treated WF, named WF-KH550.

Acetic anhydride pretreatment [34]: The dry fibers were immersed in acetic anhydride for 15 min at room temperature, and then the acetylation was carried out for 120 min at 120 °C under atmospheric pressure without a catalyst using acetic anhydride. The acetylated fibers were rinsed with water to remove the acetic acid and excess acetic anhydride until no acid smell leaked out. The WF was then dried at 60 °C to obtain acetylated WF, named WF-Ac_2_O.

### 3.3. Production of FDM Filaments

Four mixtures were first prepared, which were 5wt%WF + 95wt%PLA, 5wt%WF-KH550 + 95wt%PLA, 5wt%WF-Ac_2_O + 95wt%PLA and 5wt%WF + 90wt%PLA + 5wt%ACR, then they were pelletized, respectively using a twin-screw extruder (SHJ-20, Nanjing Giant Machinery Co. Ltd., Nanjing, China) using the process parameters listed in Table 3.

The FDM filaments of neat PLA and the aforementioned four mixtures with a diameter of 1.75 ± 0.05 mm were finally prepared using a twin-screw extruder (KS-HXY, Kunshan Huanxinyang Electrical Equipment Co. Ltd., Suzhou, China) at a temperature between 170 °C and 190 °C from hopper to die.

### 3.4. Specimens Manufacturing

A MOSHU S108 desktop-level 3D printer from Hangzhou Shining 3D Technology Co. Ltd. (Hangzhou, China) was used equipped with a 0.4 mm nozzle. The sample model file (STL file) for the test was computer-aided designed and was then sliced and transformed into G-code. Printing parameters were set as shown in Table 4.

The composite was expressed as WF/PLA when WF was not treated anyway, and the composites were labelled WF-KH550/PLA, WF-Ac_2_O/PLA and WF-ACR/PLA, respectively. When WF was treated with a coupling agent, acetic anhydride or ACR was used.

### 3.5. Mesurement and Characterization

#### 3.5.1. Fourier Transform Infrared Analysis (FTIR) of Wood Flour

Various WF was mixed and milled with potassium bromide by the mass ratio of 1:100, and then the tablet sample was prepared. The FTIR analysis was performed using Frontier spectroscopy (VERTEX 70, Bruker Optics, Ettlingen, Germany). The background was collected before any measurement. All measurements were performed with a spectrum range covering 400 cm^−1^ to 4000 cm^−1^, a scan speed of 1 cm/s and a resolution of 4 cm^−1^. A total of 32 scans were collected, and the baseline was subtracted for correction. The Bruker Spectrum software was used for the data analysis.

#### 3.5.2. Melt Index (MI)

The MI was measured according to the Chinese national standard GB/T 3682-2000 to characterize the flowability of the samples. The samples were weighed and put into the melt index meter (XNR-400, Chengde Jinhe Instrument Manufacturing Co. Ltd., Chengde, China), and the MI measurements were carried out at 180 °C and 1.26 kgf.

#### 3.5.3. Relative Die Swell Ratio

When the filament was extruded out of the die, its diameter was measured at five different points, the averaged value was recorded as its diameter after die swell and the relative die swell ratio (DS) was calculated using the following equation:

(1) $$\mathrm{DS}\;=\frac{D}{D_{0}}$$

where D_0_ is the averaged diameter of unmodified WF/PLA filament after die swell, and D is the diameter of the modified WF/PLA filament after die swell.

#### 3.5.4. Determination of Mechanical Properties

The tensile experiments were operated in accordance with ASTM D 638-14 using a universal machine (E44.304, MTS Industrial Systems (China) Co. Ltd., Shenzhen, China) at a crosshead speed of 10mm/min. The flexural experiments were operated in accordance with ASTM D 790-17 at a rate of 5 mm/min crosshead speed. The impact experiments were operated in accordance with ASTM D 256-10. The strength and modulus were evaluated as the average of at least ten specimens.

#### 3.5.5. Thermogravimetric Analysis (TGA)

The thermogravimetric analysis (TGA) of the 3D-printed specimens was performed using a TG 209F1 analyzer (NETZSCH-Gerätebau GmbH, Selb, Germany). Approximately 5mg samples were weighed and placed in an alumina ceramic crucible. Then, the TGA measurements were carried out between 25 °C and 600 °C at a heating rate of 20 K/min under a N_2_ atmosphere.

#### 3.5.6. Dynamic Mechanical Thermal Analysis (DMTA)

Dynamic mechanical thermal properties were measured in a three-point bending mode referred to literatures [6,35] using a dynamic mechanical analyzer (DMA 242C, Netzsch, Bavaria, Germany), and the properties of the samples were measured with a temperature sweep of 2 °C/min and a frequency of 5 Hz over a temperature range of 30 °C to 110 °C.

#### 3.5.7. Water Absorption

Water absorption tests were carried on the 3D-printed specimens referred to the literature [35]. After being dried 12 h in an oven at 105 °C, the specimens were weighed and later immersed in distilled water at 25 °C. Five samples were used for each composite composition. When taking readings, the samples were removed from the distilled water and blotted dry with tissue paper then weighed to the nearest 0.0001 g. The weight percentage increase *x_t_* during the immersion was calculated using the following equation:

(2) $$x_{t}=\frac{w_{t}-w_{0}}{w_{0}}\times 100\%$$

where *w*_0_ is the weight of the specimen before immersion in distilled water, and *w_t_* are the weights after immersion.

## 4. Conclusions

In this study, 3D printing WF/PLA composite materials were manufactured using fused deposition modeling (FDM) 3D printing technology, and the effects of pretreatment of WF and addition of ACR on the properties of WF/PLA were investigated. The following conclusions can be drawn from the research: 

(1) After WF pretreatment or the addition of ACR, the melt index of WF/PLA composite filament was decreased, while its melt die swell ratio increased.

(2) Coupling agent pretreatment on WF, acetic anhydride pretreatment on WF and the addition of ACR were all shown to improve the overall mechanical performance of the composites, and the addition of ACR exhibited the most apparent improvement in the mechanical properties; it increased the tensile modulus, flexural strength, flexural modulus and impact strength by 13.83%, 22.11%, 27.56% and 35.17%, respectively, more than those of WF/PLA.

(3) The thermal decomposition temperature and the char residue after the thermal composition of WF/PLA increased as a result of the WF pretreatment and addition of ACR.

(4) The storage modulus at 30 °C of WF/PLA was increased by 6.50%, 13.11% and 12.25%, respectively, after KH550 pretreatment on WF, Ac_2_O pretreatment on WF and the addition of ACR. All the modified composites had slightly higher T_g_ values than virgin WF/PLA. Among all the composites, WF-ACR/PLA had the highest T_g_ of 70.70 °C.

(5) All the modified composites absorbed less water than virgin WF/PLA at any immersion duration, and Ac_2_O pretreatment on WF and the addition of ACR can lower the water absorption of the printed specimens more obviously than that by KH550 pretreatment on WF.

The present study emphasizes the effects of chemical modification on the mechanical, thermal, dynamic mechanical thermal and water uptake properties of WF /PLA; however, there is still a lack of information on the relationship between the printing parameters (FDM) and the properties of chemical-modified WF/PLA. In addition, as a theoretically biodegradable composite, the effects of the chemical modification on the degradation properties of WF/PLA have not been investigated. We still require the optimization of the printing parameters to improve the performance of the modified WF/PLA, and we must conduct further research to make the relationships between the chemical modification and the degradation behaviors of WF/PLA clear.

## Figures and Tables

**Table 1:** Thermal decomposition parameters for various composites in a nitrogen atmosphere.

Specimen	T_i_/°C	T_P_/°C	Weight Residue at 600 °C/%
WF/PLA	307.8	356.1	0.46
WF-KH550/PLA	314.2	355.6	0.54
WF-Ac_2_O/PLA	321.8	360.0	0.55
WF-ACR/PLA	319.4	357.8	0.58
PLA	334.5	375.8	0.19

**Table 2:** Tg of unmodified and modified PLA/WF composites (Frequency: 5 Hz).

Specimen	WF/PLA	WF-KH550/PLA	WF-Ac_2_O/PLA	WF-ACR/PLA
T_g_	68.20	69.22	70.70	68.95

Note: Tg is the temperature range where the polymer substrate changes from a rigid glassy material to a soft (not melted) material.

**Table 3:** Twin-screw extruder process parameters.

Parameter	Temperature of Zone I/°C	Temperature of Zone II/°C	Temperature of Zone III/°C	Temperature of Zone IV/°C	Rotating Speed /(r/min)
value	160	170	165	155	75

**Table 4:** Three-dimensional Printing Process Parameters.

Parameter	Layer Thickness /mm	Temperature of Nozzle /°C	Temperature of Bed /°C	Printing Speed/ mm/s	Printing Angle /°
value	0.2	220	55	40	0

## Data Availability

Not applicable.

## Sample Availability

Samples of the compounds are not available from the authors.
